# Supplementary material for: Structural and functional insights of the human peroxisomal ABC transporter ALDP
Source: eLife. 2022 Nov 14;11:e75039. doi: 10.7554/eLife.75039 (PMC9683791; doi:10.7554/eLife.75039)
Supplement: Figure 1—source data 1. [file elife-75039-fig1-data1.doc]

**Table S1. Cryo-EM data collection, refinement and validation statistics**

| **Coordinates** | 7VR1; EMD-32096 |
| --- | --- |
| **Data collection and processing** | ALDP |
| Magnification | 64,000 |
| Voltage (kV) | 300 |
| Electron exposure (e–/Å^2^) | 50 |
| Defocus range (μm) | -1.5 ~ -1.8 |
| Pixel size (Å) | 1.0979 |
| Symmetry imposed | C2 |
| Initial particle images (no.) | 1,259,972 |
| Final particle images (no.) | 135,662 |
| Map resolution (Å)  FSC threshold | 3.4  0.143 |
| Map resolution range (Å) | 3.0-4.0 |
| **Refinement** |  |
| Model composition  Non-hydrogen atoms  Protein residues  Ligands | 9,522  1,190  0 |
| R.m.s. deviations  Bond lengths (Å)  Bond angles (°) | 0.002  0.558 |
| Validation  MolProbity score  Clashscore  Poor rotamers (%) | 1.78  7.15  0.00 |
| Ramachandran plot  Favored (%)  Allowed (%)  Disallowed (%) | 94.26  5.74  0.00 |
