# Supplementary material for: Structural and functional insights of the human peroxisomal ABC transporter ALDP
Source: eLife. 2022 Nov 14;11:e75039. doi: 10.7554/eLife.75039 (PMC9683791; doi:10.7554/eLife.75039)
Supplement: Figure 3—figure supplement 3—source data 1. [file elife-75039-fig3-figsupp3-data1.docx]

**Figure 3 - figure supplement 3 - source data 1**


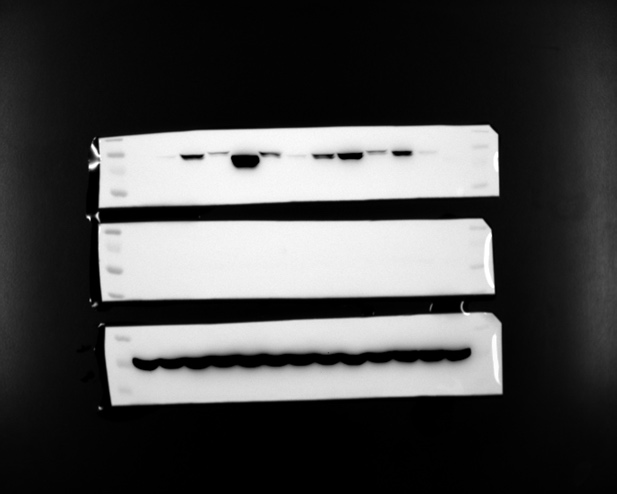

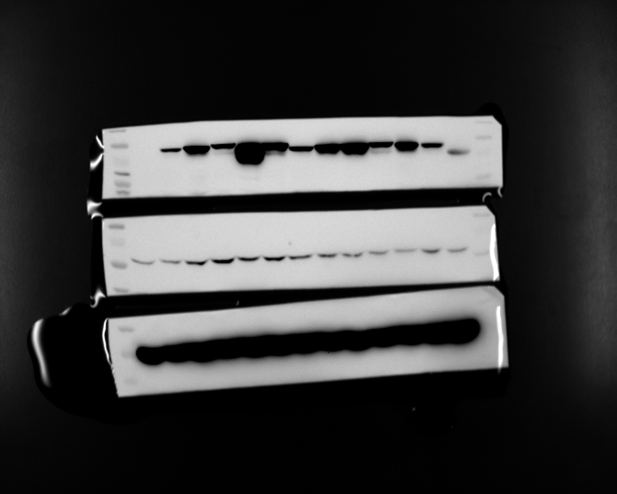


Figure 3 - figure supplement 3 - source data 1 Uncropped version of the Western blot. The overexpressed ALDP and mutation were confirmed by western blot. The upper band is anti-ALDP, the middle band is anti-Catalse, and the lower band is anti-GAPDH. The transfer results of anti-ALDP and anti-GAPDH from the same gel. GAPDH was used as loading control. Exposure time was 10min (left) and 30min(right).
